# Development of an admission criterion framework for high-cost medical consumables in governmental hospitals: a systematic review

**DOI:** 10.1186/s12913-024-11318-z

**Published:** 2024-07-29

**Authors:** Tianshu Chu, Yahui Han, Haiyin Wang

**Affiliations:** 1 School of Public Health, Fudan University, Shanghai, China; 2 China Pharmaceutical University, Nanjing, China; 3 Shanghai Health Development Research Center (Shanghai Medical Information Center), Shanghai, China; 4 National Health Commission Key Laboratory of Health Technology Assessment (Fudan University), Shanghai, China

**Keywords:** High-cost medical consumables, Governmental hospital, Admission criterion, Purchasing management, Systematic review

## Abstract

**Background:**

High-cost medical consumables (HMCs) have emerged as significant economic and technological challenges for numerous national healthcare systems. Governmental hospitals play an indispensable role in many national health systems, closely linked to the evaluation of admissions and the management of procurement for HMCs. Nevertheless, many governmental hospitals face avoidable management risks due to the lack of a decision-making tool. In response, we conducted a systematic review to establishing a framework for the admission criteria of HMCs. This framework aims to enhance their effective utilization and maximize economic, clinical, and social benefits.

**Methods:**

In accordance with a systematic review protocol developed for our study, we conducted comprehensive searches in the PubMed, Web of Science, and Embase databases to identify all correlation studies conducted prior to December 31, 2021. Subsequently, two independent reviewers performed a two-round screening process, resulting in the inclusion of 23 articles in our study. Finally, a third reviewer meticulously examined the selected indicators and contributed to the development of the final criterion framework.

**Results:**

The criterion framework was established with 7 first-level indicators and 23 s-level indicators. Among the first-level indicators, "Clinical Benefit" held the highest significance, with a combined weight of 1.606, followed by "Economic Value" and "Organizational Impact" at 1.497 and 1.159, respectively. At the second level, "Safety" and "Efficacy" carried equal weight in the decision-making tool, with combined weights of approximately 1.300 each and a standard combined weight of 0.130.

**Conclusion:**

This admission criteria framework serves as a vital decision-making tool for managing admissions and highlights several crucial evaluation indicators. Economic considerations emerge as the principal determinant in HMCs procurement decisions. Consequently, healthcare managers and decision-makers are recommended to give precedence to value-based healthcare and evidence-based procurement practices. In the long term, governmental hospitals must grapple with the challenge of judiciously allocating limited resources to maximize both social and economic benefits.

## Background

High-cost medical consumables (HMCs) are defined as medical consumables that have a direct impact on human health, have stringent safety regulations, are in widespread clinical use, with a relatively high price and significant financial burden on the general public [1]. HMCs generally encompass specialized medical treatment materials, including cardiac intervention devices, peripheral vascular intervention equipment, artificial joints, and other alternative organ-related medical supplies. HMCs necessitate stringent safety controls and are restricted for specific applications [2, 3]. Furthermore, given their complex technology, diverse categories, and frequent updates, the effective and rational utilization of HMCs holds profound implications for medical safety, quality, technological advancements, and economic and societal benefits [4, 5]. A resolution adopted during the 2007 World Health Assembly underscored the economic and technological challenges posed by health technologies and medical devices, including HMCs, for numerous national healthcare systems [6]. Naturally, advanced technologies typically always come with higher prices compared to their ordinary or conventional counterparts, thereby imposing a significant financial burden on healthcare systems and patients [7]. While HMCs play a pivotal role in advancing clinical technology and disciplines, their high costs and substantial clinical demands have led to the inefficient utilization of scarce medical resources. This issue has now taken center stage in national medical insurance governance deliberations [8, 9].

The development of a rapid, scientifically sound, and appropriate tool for guiding admission decisions regarding HMCs has been a prominent area of research interest. In European nations such as England, France, and Germany, the procurement of HMCs has historically been driven primarily by considerations of price and cost, possibly overlooking a broader spectrum of equity and social criteria. This implies that decision-makers should shift their focus towards the effectiveness, social benefits, and other values that HMCs offer to patients and healthcare providers, rather than solely concentrating on price and cost control. In contrast to traditional cost-centered procurement practices, adopting an evidence-based procurement approach could help eliminate inefficient treatments and foster innovation in advanced medical technologies. Through increased adoption of evidence-based procurement, countries like England and others may facilitate a transition in admission strategies, moving from a cost-centered approach to one that is value-centered. Health Technology Assessment (HTA) is a multidisciplinary process that uses explicit methods to determine the value of a health technology at different points in its lifecycle. The purpose is to inform decision-making in order to promote an equitable, efficient, and high-quality health system [10]. With the development and application of HTA, an innovative and cutting-edge viewpoint on the value-based healthcare or procurement has gained a lot of attention, emphasizing the effectiveness of cost, clinical benefit, and social impact. This shift can pave the way for the establishment of a value-based pricing system, incorporating HTA methodologies to maximize the economic and social benefits derived from HMCs [11].

The Chinese government has also recognized the importance of reforming the management of HMCs, characterized by their high unit prices and resource consumption. To standardize medical service practices and control the unreasonable growth of medical expenses, the income generated from HMCs will be included in the performance evaluations of governmental hospitals. Additionally, apart from a select few specialized medical consumables, a broad spectrum of HMCs will either be independently covered by medical insurance or incorporated into the list of covered medical services. Furthermore, the development of an admission management system, an admission product catalog, and a dynamic adjustment mechanism for medical consumables is underway, with regular updates planned [12]. However, as the reform process progressed, challenges began to emerge due to the absence of a scientific admission mechanism and decision support tool for HMCs in the majority of governmental hospitals. Numerous prevalent issues included a lack of an efficient management framework, non-transparent admission processes, and a dearth of effective management tools, all of which escalated management risks [13–15].

For the management practice in China, governmental hospitals still played a major role in the procurement and admission of HMCs, in addition to the current province-organized procurement of coronary stents, orthopedics and other varieties, as well as the alliance procurement carried out by provinces and cities [16]. Hence, it became imperative to explore an admission decision-making tool for HMCs in governmental hospitals to mitigate unreasonable admission management risks. A few governmental hospitals in China had begun to explore the introduction of hospital-based HTA and the construction of corresponding decision-making tools. Hospital-based HTA was a systematic and comprehensive evaluation of the short-term and long-term impacts of health technology on the safety, efficacy, and economy of health technology in hospital scenarios, and this method had gradually been used as an important theoretical basis for decision-making tools for HMCs admission in governmental hospitals [17]. For instance, Professor Qian Xiang suggested a tool for demand assessment, technical assessment, and economic evaluation of equipment and consumables to be purchased based on the hospital-based HTA process. Then, he prioritized equipment purchases in accordance with the hospital budget [18]. Professor Xuelian Peng proposed that the access of medical consumables should be evaluated according to five dimensions (safety, efficacy, economy, cooperation, and social adaptability), and each dimension should be screened and evaluated step by step [19].

In our study, aiming to gain insights from international experiences in admission evaluation and procurement management of HMCs, we conducted a systematic review following a standardized protocol. We comprehensively gathered all relevant indices to develop an admission criterion framework for HMCs within governmental hospitals. This framework would be poised to enhance the efficient allocation of medical resources and augment their economic, clinical, and societal benefits within governmental healthcare institutions.

## Method

In accordance with the guidelines outlined on the PRISMA website, we meticulously developed and adhered to a systematic review protocol for our study [20, 21]. This protocol was autonomously formulated and primarily comprised: defining the research question, delineating the search strategy, establishing selection criteria encompassing both inclusion and exclusion criteria, designing a data collection form, and conducting a comprehensive two-phase review process. In the initial phase, two reviewers independently screened titles and abstracts, and subsequently, retrieved full texts and applied the predefined selection criteria [22].

### Search strategy

For our study, we conducted comprehensive searches in the PubMed, Web of Science (WOS), and Embase databases to collect all relevant studies conducted prior to December 31st, 2021. We exclusively focused on open-access publications and restricted our search to papers. The search process involved manual retrieval and the application of keywords within the titles, abstracts, and body text. The keywords were organized into four distinct sections, with many of them referencing MeSH terms. The first section pertained to "high-cost" or "high-value," while the second section centered on "medical consumables" and included various associated keywords such as "Technology," "Device," "Consumables," "Equipment," "Supplies," and "Instrumentation." The third section focused on "admission studies" and encompassed associated keywords like "Access," "Admit," "Adoption," "Procurement," and "Approval." Lastly, the fourth section centered on "hospital" and incorporated additional keywords like "Administration," "Decision Making," "Organizational," and "Decision Support Techniques." Within each section, we utilized logical operators like "OR" to combine keywords, and then we used "AND" to combine queries from each section, resulting in a comprehensive and highly specific search query.

### Selection criteria

Following the removal of duplicate records, a rigorous screening process was conducted by two independent reviewers. Recorder 1 (HYH) is a postgraduate student at China Pharmaceutical University, while Recorder 2 (CTS) is a doctoral student from the School of Public Health, Fudan University. In a comprehensive two-round screening procedure, both reviewers meticulously assessed the titles, abstracts, and full-text content of the papers to ascertain their alignment with the predefined selection criteria.

#### Inclusion criteria

Papers eligible for inclusion in this study must meet the following criteria:

(a) They should be Health Technology Assessment (HTA) studies.

(b) They should pertain to the development of decision-making tools.

(c) They should employ Multi-Criteria Decision Analysis (MCDA), which is an extension of Multiple-objective decision-making theory, often integrating multi-objective guidelines as a whole to rank health technologies through a range of methods to determine the best choice [23].

(d) The research should be conducted from the hospital perspective.

#### Exclusion criteria

Papers meeting any of the following criteria were excluded from consideration:

(a) They are systematic review studies.

(b) They focus on a single research object or have inconsistent research objects.

(c) The studies lack a criterion framework.

(d) The research is conducted from a governmental perspective.

### Data collection and extraction

Given that our study primarily centered on qualitative data encompassing parameters, indicators, and criteria, we independently devised a data collection form. This form included fields for recording the year of publication, authorship details, country of origin, characteristics of the study objects, research methods, criteria, and other relevant variables. When developing our criterion framework for HMCs, the majority of included articles often provided a two-level criterion framework. Taking operability and application into consideration, we decided to adhere to the structure of framework commonly used in the literature. First, we chose two or three articles as the core draft, which had a comprehensive structure and common indicators. Second, additional indicators addressed in other studies were added to the basic draft, and indicators with similar meanings were matched wherever feasible. Thirdly, reviewers would have a group discussion, and remove or combine the indicators that did not conform to the requirements. Two reviewers meticulously gathered data from the selected studies and collaboratively constructed an initial criterion framework. Subsequently, a third reviewer, identified as WHY, was enlisted to scrutinize the initial criterion framework. This involved deliberations regarding criteria for inclusion or exclusion and the consolidation of duplicates to formulate the final criterion framework.

### Data analysis

To quantify the contribution of each indicator to our admission criterion framework, we had adopted the calculating method of combining weights in the analytic hierarchy process (AHP) and simplified it for our study [24]. We evaluated the combined weight of each indicator based on two factors: the Journal Impact Factor (IF) and the quantity of supporting evidence. For a given indicator "i", the IF score ("score_i") was determined by aggregating the IF values of the supporting articles. Subsequently, each IF score ("score_i") was normalized to yield the IF weight ("weight_i") using the following formula:

$${Weight}_{i}= \frac{{Score}_{i} }{{Score}_{max}}$$

Similarly, the score for quantity was determined by summing the number of supporting articles. Each score for quantity was then rescaled to the weight of quantity. Then, the combined weight was the sum of the weights for IF and quantity, and it was also rescaled to the standard combined weight using the above formula.

## Results

### Search and study selection

**Fig. 1:**
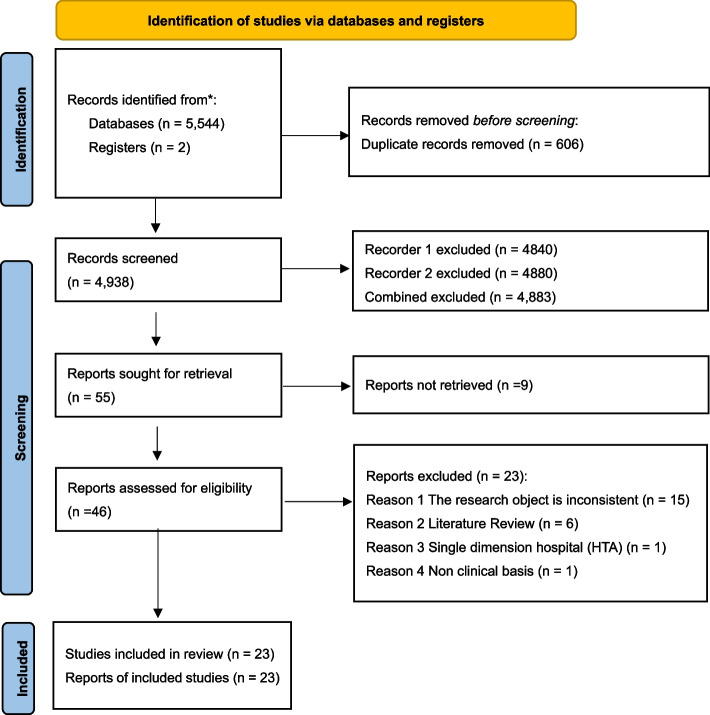
Flow chart of evidence selection

A total of 5,544 literature records were generated during the initial literature search for our study. This comprised 1,716 papers from PubMed, 3,338 papers from Web of Science (WOS), and 490 papers from Embase. Before the screening process commenced, we conducted a meticulous manual cross-check of the records using Endnote 20.0, resulting in the removal of duplicate records (n = 606). This left us with a total of 4,938 unique articles for further assessment. In the first round of screening, two independent reviewers, namely HYH and CTS, evaluated each manuscript primarily based on its title and abstract, guided by the predefined selection criteria. Recorder HYH excluded 4,840 papers, while Recorder CTS excluded 4,880 papers, with a total of 4,883 papers excluded due to overlapping decisions. Subsequently, full-text versions of 55 articles were retrieved for further examination, but 9 articles had to be disqualified as we were unable to access them. In the second round of screening, both reviewers assessed each paper predominantly based on its full-text content. Following independent evaluations by the two reviewers, 23 papers were removed from our analysis. These exclusions were attributed to various reasons, including inconsistencies in the research object (15 papers), literature review focus (6 papers), single-dimension hospital focus (1 paper), and a lack of clinical basis (1 paper). Ultimately, our study included a total of 23 articles. The comprehensive flowchart illustrating the outcomes of the evidence selection process is presented in Fig. 1.

### General characteristics of included studies

Among the 23 papers included in our study, the publication years spanned from 2012 to 2022. These papers originated primarily from various regions, including Europe (e.g., Italy, Germany, and France), Asia (e.g., China and India), and North American countries (e.g., the United States and Canada). Notably, four of the included studies conducted transnational research efforts. The predominant methodologies employed in these papers encompassed expert interviews, questionnaires, and HTA analyses. When considering the Journal Impact Factor (IF) of the articles, we found that the average IF among the papers was 3.124, with the highest IF being 8.000.

### Admission criterion framework of HMCs

The two-level admission criterion framework for HMCs comprised seven first-level indicators and twenty-three second-level indicators. Among the first-level indicators, "Clinical Benefit" emerged as the most significant parameter, boasting a combined weight of 1.606, surpassing all other indicators. Following closely were "Economic Value" and "Organizational Impact" with combined weights of 1.497 and 1.159, respectively. These three parameters, "Clinical Benefit," "Economic Value," and "Organization," represented the primary criteria at the first level, with a total standard combined weights of approximately 0.600. Conversely, "Social Impact" and "Clinical Needs" carried combined weights lower than 0.500, with their standard combined weights hovering around a mere 0.070.

**Fig. 2:**
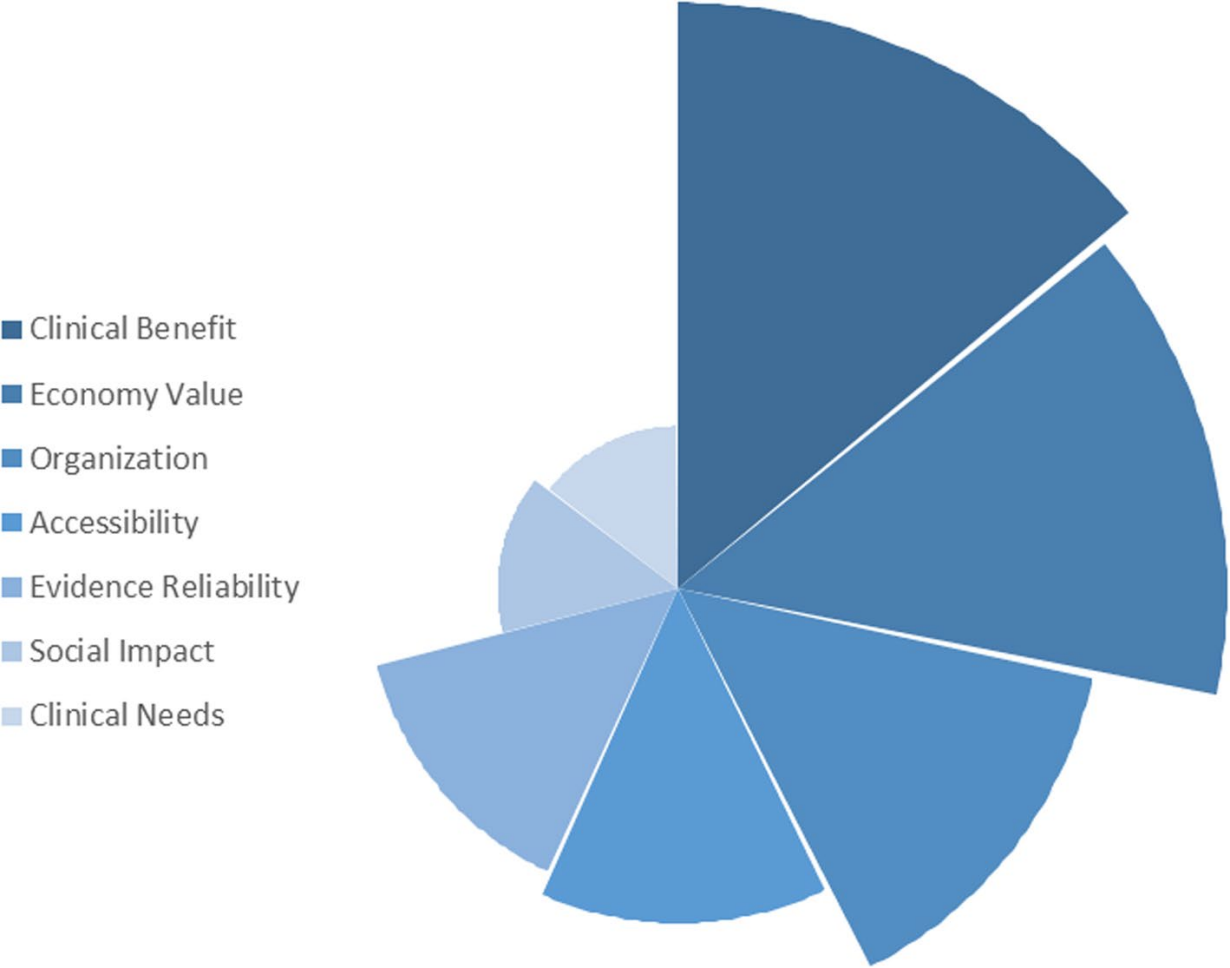
Diagram about the standard combined weight of the first level indicators

**Fig. 3:**
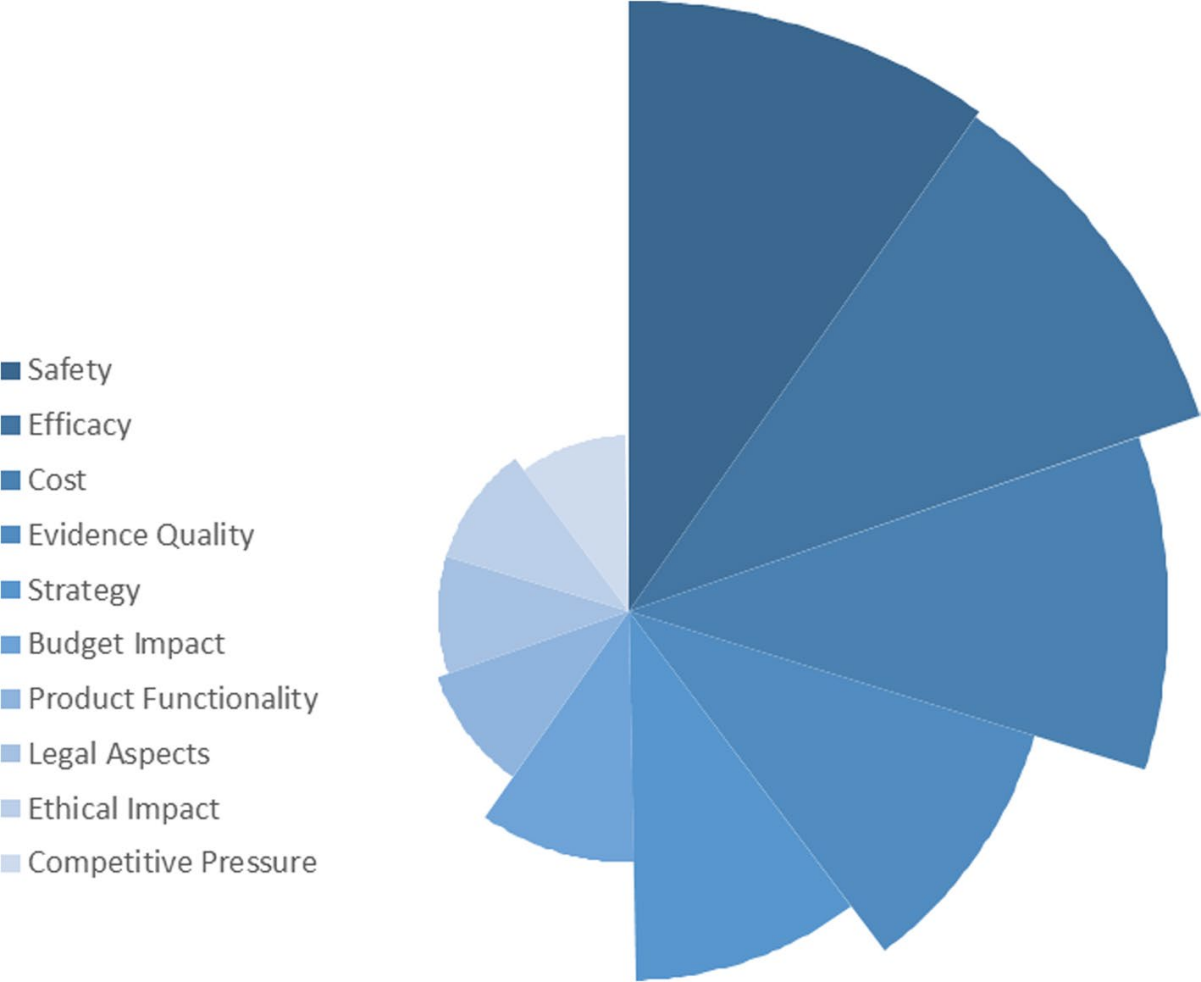
Diagram about the top 10 standard combined weight of the second level indicators

Regarding the second-level indicators, "Safety" and "Efficacy" made nearly equal contributions, with combined weights of approximately 1.300 and standard combined weights of about 0.130, ranking them higher than any other indicators. Following closely, "Cost" secured the third position, with a combined weight and standard combined weight of 1.180 and 0.116, respectively. Subsequently, "Evidence Quality" and "Strategy" exhibited combined weights slightly below 1.000, accompanied by standard combined weights of 0.091 and 0.079, respectively. These five parameters, namely "Safety," "Efficacy," "Cost," "Evidence Quality," and "Strategy," constituted the primary contributors within the second-level framework, with a total standard combined weight exceeding 0.500. In contrast, the "Transition," "Support/Implementation," "Gross Domestic Product (GDP)," and "Epidemiological Burden" all performed with combined weight lower than 0.100, and standard combined weight below 0.010. Comprehensive details are provided in Table 1, Figs. 2 and 3.

**Table 1:** Admission criterion framework and indicator weight of HMCs

First Level Indicators	Weight of IF	Weight of Quantity	Combined Weight	Standard Combined Weight	Second Level Indicators	Weight of IF	Weight of Quantity	Combined Weight	Standard Combined Weight
Social Impact	0.234	0.261	0.495	0.071	Legal Aspects	0.201	0.217	0.418	0.041
					Ethical Impact	0.201	0.217	0.418	0.041
					Equity	0.033	0.043	0.077	0.008
Clinical Needs	0.229	0.217	0.447	0.064	Patient Demand	0.100	0.087	0.187	0.018
					Epidemiological Burden	0.017	0.043	0.060	0.006
					Economic Burden	0.050	0.087	0.137	0.013
					Severity of the Disease	0.079	0.087	0.166	0.016
Organization	0.594	0.565	1.159	0.166	Revenue Factors	0.078	0.043	0.121	0.012
					Strategy	0.416	0.391	0.807	0.079
					Competitive Pressure	0.212	0.174	0.386	0.038
Clinical Benefit	0.824	0.783	1.606	0.230	Safety	0.685	0.652	1.337	0.131
					Efficacy	0.670	0.652	1.322	0.130
					Public Health Benefits	0.067	0.087	0.154	0.015
Economy Value	0.758	0.739	1.497	0.215	Cost	0.615	0.565	1.180	0.116
					Budget Impact	0.288	0.261	0.549	0.054
					Cost-effectiveness	0.143	0.174	0.317	0.031
Evidence Reliability	0.461	0.391	0.852	0.122	Evidence Quality	0.494	0.435	0.929	0.091
					Evidence Quantity	0.212	0.174	0.386	0.038
Accessibility	0.478	0.435	0.913	0.131	Brand Effect (Reputation)	0.157	0.130	0.287	0.028
					Service Quality	0.198	0.174	0.372	0.036
					Transition Support/Implementation	0.033	0.043	0.077	0.008
					Product Functionality	0.226	0.217	0.443	0.043
					Gross Domestic Product (GDP)	0.033	0.043	0.077	0.008

## Discussion

According to existing research, while the evaluation dimension of hospital-based HTA was in line with the actual management of HMCs, comprehensive evaluation results had not been formed. Our study developed a decision-making tool for the admission of HMCs appropriate for governmental hospitals based on hospital-based HTA procedures and systematic reviews, which had significant theoretical and practical application value: a) The decision-making tool could be used by hospital administrators to enhance the scientific and efficient admission of HMCs, particularly for hospitals in low- and middle-income countries, to assist in determining whether to admit HMCs or which HMCs should be admitted in multiple dimensions. b) Helped governmental hospitals comprehend the admission requirements of HMCs and improve the admission process for HMCs. c) Extended the current theoretical frameworks for the decision-making tool for HMCs in governmental hospitals. Furthermore, we would follow the NICE (National Institute for Health and Care Excellence) methods or EUnetHTA core model, and also consider the MCDA theoretical approach to describe the weights of different criterion dimensions and provide a comprehensive visual score. Additionally, we also identified some indicators worth discussing in our criterion framework.

### Economic reason was the core of HMCs admission management

"Economic Value" emerged as one of the most pivotal criteria among the first-level indicators, underlining the imperative consideration of economic factors in the management of admission and procurement of new HMCs. Specifically, economic considerations had a pronounced impact in two significant dimensions. One was the pressure exerted by costs, and the other revolved around the feasibility of acquiring new HMCs within the existing economic context. The cost factor perennially held primacy in the realm of HMCs admission management, intricately intertwined with the budgetary considerations of governmental hospitals. Consequently, cost-effectiveness assumed a paramount position in the organizational priorities of governmental healthcare institutions. The financial burden, especially concerning products with limited lifespans and single-use consumables, necessitated careful evaluation by decision-makers. This evaluation encompassed the assessment of procurement's implications for the overall annual hospital budget and the economic or clinical benefits that HMCs could offer to both healthcare providers and patients [25, 26]. It implied that HMCs would experience different competitive pressures from accessible alternatives and would be compared with one another to identify the optimal products. In other words, a more friendly price and highlights of clinical benefit could attract the mangers from governmental hospitals. Additionally, accessible HMCs could help the majority of patients receive high-quality medical treatment and access to the benefits from the development of advanced medical devices, which were the requirements for medical equity.

In the latter context, our study shed light on an unconventional perspective regarding the criterion, specifically focusing on the economic climate explanation, which conventionally correlates with GDP and revenue. To delve deeper, the economic climate encompassed the capacity of healthcare providers to invest and the financial accessibility for patients. In China, governmental hospitals predominantly relied on funding from the government and medical insurance funds, both subject to stringent regulation by the local health ministry and bound by local GDP constraints. Simultaneously, the primary payment methods, namely urban workers' insurance and rural and urban resident basic medical insurance, exhibited close ties to local revenue levels. These revenue dynamics directly influenced the affordability of the majority of patients. Consequently, particularly for governmental hospitals heavily reliant on local revenue streams, the economic climate stood as the decisive factor influencing the procurement of HMCs [27, 28]. In addition to the aforementioned factors, the World Health Organization (WHO) has consistently championed efforts to enhance the accessibility of primary healthcare services. The concept of Universal Health Coverage (UHC), spearheaded by WHO, underscores the imperative for governments to establish accessible, robust, and patient-centric primary healthcare systems deeply embedded within local communities [29]. It has been recognized that establishing a strong foundation for advanced healthcare services begins with meeting the essential requirements of basic medical care. This principle is particularly salient in low and middle-income regions, where the demand for advanced medical services tends to be comparatively lower, and many patients may face challenges in affording HMCs. In such contexts, local hospitals often prioritize social impact considerations over clinical effectiveness, striving to equitably distribute their limited medical resources. Excessive allocation of medical resources to HMCs could yield limited economic and social benefits, potentially exacerbating issues of medical inequality and misaligning with the objectives of UHC. In essence, the procurement of HMCs should be aligned with the strategic development plans of hospitals and the prevailing stage of economic development.

### Balance the conflict between clinical benefit and clinical needs

Within the first-level indicators, "Clinical Benefit" held the highest combined weight among all criteria. It is noteworthy that two second-level indicators, namely "Safety" and "Efficacy," both integral components of "Clinical Benefit," made equitable and substantial contributions to the procurement and management of HMCs. These two criteria stood out as the most pivotal considerations within the admission criterion framework. Conversely, "Clinical Need" and its corresponding second-level criteria consistently exhibited combined weights lower than other factors. Notably, safety has remained a perennial and paramount concern throughout the evolution of medicine and healthcare, driven by the imperative to avert medical mishaps. Numerous studies have characterized safety concerns as potential adverse effects on patients associated with the introduction of new HMCs. Clinical risk management, reflecting the capacity to control and mitigate adverse events, has been a key tool in evaluating the safety of emerging medical technologies [30, 31]. Efficacy has garnered increasing attention, particularly in light of the advancements in HTA. HTA has proven instrumental in comprehending the cost-effectiveness of HMCs. Concurrently, it has become apparent that, relative to other indicators, "Clinical Needs" and its corresponding second-level criteria hold a relatively lower degree of significance within the admission criterion framework.

In more precise terms, the conflict between "Clinical Benefit" and "Clinical Needs" represents a clash between two distinct justifications. On one hand, value-based healthcare emphasizes the efficient allocation of medical resources to maximize clinical benefit and social impact, avoiding unnecessary medical interventions and alleviating the disease burden among patients [32, 33]. Health-centered care often aligns more closely with the core values of the medical profession. Physicians strive to use their knowledge, skills, and resources to enhance individual and public health. However, the finite nature of medical resources and supply capacity means not all patient needs can be met. While health-centered care promotes improved health outcomes, it can also lead to higher costs and potential overuse of medical services. This challenge is especially pronounced in low-income areas, where the equitable allocation of limited medical resources is a persistent issue. Thus, in procuring HMCs, the priority is given to "Clinical Benefit" over "Clinical Needs," driven by budgetary constraints. As we manage cost control in HMC procurement, addressing health-related social needs becomes crucial in pursuing medical equity.

### Evidence, legal and ethical issues are the trigger condition

Similar to other medical services and technologies, the fundamental criterion for evaluating new HMCs is the reliability of evidence. The quality and quantity of medical evidence are widely-accepted parameters to assess whether new medical technology can be integrated into clinical practice. As evidence-based medicine advances, a standardized evaluation system emerges to facilitate the generation of high-quality medical evidence, thereby mitigating safety concerns. Additionally, a range of social impact factors garners increasing attention, notably legal and ethical considerations. Ethical and moral concerns, particularly surrounding the use of artificial organs, necessitate stringent legal constraints. Consequently, the rigorous scrutiny of experiments demands ethical statements to standardize medical research. In summary, the aforementioned criteria constitute the threshold conditions for the admission management of HMCs. Products lacking sufficient clinical evidence or entangled in legal or ethical issues are subject to direct exclusion from the procurement process.

### Limitation

Our systematic review focused primarily on qualitative research, limiting our ability to conduct a meta-analysis to enhance the overall quality of evidence. The combined weight, derived from factors such as the Impact Factor of journals and the number of studies, still fell short of constituting a robust body of evidence, resulting in the exclusion of some reports published on agency websites. Additionally, presenting assessments of certainty or confidence in the evidence and reporting on the risk of bias proved challenging. To address these limitations, we are preparing a two-round expert consultation using the Delphi method. Furthermore, we intend to employ the Analytic Hierarchy Process method to enhance the quality of our admission criteria framework.

## Conclusion

Governmental hospitals serve as foundational components of many national healthcare systems and major providers of medical services. In their major role in the procurement and access of HMCs, the absence of a rational and accessible decision-making tool has consistently exposed governmental hospitals to management risks. The adoption of irrational management techniques and strategies for HMCs can precipitate significant challenges, including medical inequality and the wastage of valuable medical resources. Such mismanagement can diminish the economic benefits and societal impacts these advanced technologies offer to patients. In light of these challenges, our study conducted a systematic review to construct an admission criterion framework for HMCs. This framework serves as a valuable decision-making tool for procurement within governmental hospitals, enhancing the efficiency and effectiveness of HMC utilization while safeguarding economic, clinical, and societal benefits for patients.

We constructed a two-level criterion framework for HMCs, comprising seven first-level indicators and twenty-three second-level indicators. At the first level, "Clinical Benefit" emerged as the most pivotal parameter, followed closely by "Economic Value" and "Organization." These three parameters assume a paramount role as major criteria, whereas "Clinical Needs" holds relatively lower significance. At the second level, "Safety" and "Efficacy" made substantial contributions to the criterion framework, with their combined weight surpassing any other factors. "Cost" occupied the third position, followed by "Evidence Quality" and "Strategy." These five parameters emerge as the primary contributors to our framework. In sum, economic considerations are central to the procurement process for HMCs, reflecting the substantial financial burden associated with these products. This underscores the imperative of adopting value-based healthcare and evidence-based procurement as primary approaches within governmental hospitals. Achieving the rational utilization of medical resources to maximize societal and economic benefits remains an enduring challenge for healthcare systems.

## Data Availability

The datasets used and/or analysed during the current study are available from the corresponding author on reasonable request.
